# Multi-functional gene ZNF281 identified as a molecular biomarker in soft tissue regeneration and pan-cancer progression

**DOI:** 10.3389/fgene.2022.1082654

**Published:** 2023-01-05

**Authors:** Xueying Hou, Jie Luan, Su Fu

**Affiliations:** Breast Plastic and Reconstructive Surgery Center, Plastic Surgery Hospital, Chinese Academy of Medical Sciences, Peking Union Medical College, Beijing, China

**Keywords:** ZNF281, regeneration, tumorigenesis, biomarker, pan-cancer

## Abstract

Regeneration and tumorigenesis are indicated as related processes, while regeneration leads to life and the outcome of tumorigenesis is death. Here, we show the upregulation of *zfp281* (zinc finger 281) in our adipose *de novo* regeneration model through RNA-seq analysis. Then, we validated the upregulation of zfp281 in adipose regeneration *via* immunofluorescence. Following that, we found that *ZNF281* (the human homolog of *Zfp281*) was upregulated in most types of cancer and related to worse prognosis in 10 tumors. We further investigated the role of *ZNF281* in cervical squamous cell carcinoma and endocervical adenocarcinoma (CESC), pancreatic adenocarcinoma (PAAD), and stomach adenocarcinoma (STAD) and confirmed the high accuracy in the clinical diagnostic feature. Beyond that, based on these three types of cancers, we analyzed the *ZNF281*-related tumor immune infiltration and DNA methylation sites and finally built risk prediction models for future disease diagnosis. Taken together, our findings provide new insights into the dual role of *ZNF281*, and we found that it was a potential biomarker for regeneration and tumor prognosis.

## 1 Introduction

Regeneration is characterized by the process of restoring homeostasis when organs sense the signals of damage, and this renewal process is orchestrated by a complex network of gene regulation and cellular processes (Chargé and Rudnicki, 2004; Gurtner et al., 2008; Gray et al., 2018). Like wound healing, regeneration proceeds through several overlying statuses, including inflammation (Eming et al., 2017), tissue reconstruction (Ghuman et al., 2016), and remodeling (Kim et al., 2018). All along the regeneration process, specific signals induce cellular proliferation in a finite number of cells, and finally, termination signals are released to avoid dysregulated proliferation, which causes tumorigenesis. Interestingly, increasing evidence indicates that regeneration and tumorigenesis are regulated by the same molecular pathways, and they may be recognized as related processes (Beachy et al., 2004; Schäfer and Werner, 2008; Cernaro et al., 2012; Jung et al., 2021). Thus, it is intriguing and significant to understand the link between regeneration and tumorigenesis.

ZNF281 (zinc finger protein 281) plays a role in the regulation of embryonic stem cell (ESC) differentiation and is very important in maintaining cellular stemness (An integrated encyclopedia of DNA, 2012; Pieraccioli et al., 2018). Knock-out of ZNF281 induces multipotent stem cell differentiation to osteogenic lineage (Seo et al., 2013). Moreover, epithelial-to-mesenchymal transition (EMT) is activated by ZNF281 in colon cancer cells through the regulation of SNAI1 and other EMT-related gene expressions (Hahn et al., 2013). We identified transcription factor *zfp28*1, the human homolog of which is *ZNF281*, upregulated in a mice adipose regeneration model through RNA-seq. However, the role of ZNF281 in the regeneration program and tumorigenesis is largely unveiled.

In this study, we present findings that implicate ZNF281 as a promising biomarker in regeneration and multiple cancers. We applied basic fibroblast growth factor (bFGF) *via* controlled release of decellularized cells to induce adipocyte regeneration in C57BL/6N mice, and this model has been proven efficient in inducing adipogenesis (Zhang et al., 2016). In addition to mRNA upregulation of *zfp281*, histological analysis also validated the higher expression of zfp281 in the regeneration group. Furthermore, for a more complex and systematic understanding of *ZNF281* in tumors, the expression level of *ZNF281* was also investigated in pan-cancer and an upregulated phenomenon in most tumors compared to their related normal tissues and adjacent tissues was observed. Next, *ZNF281* expression was found in 10 types of human cancer related to worse prognosis considering overall survival (OS); however, in kidney renal papillary cell carcinoma (KIRP), higher *ZNF281* expression was related to a better prognosis, and this may due to the complexity and heterogeneity in cancer. Then, in three types of cancer, cervical squamous cell carcinoma and endocervical adenocarcinoma (CESC), pancreatic adenocarcinoma (PAAD), and stomach adenocarcinoma (STAD), *ZNF281* was confirmed as a good prognostic molecular marker considering the high accuracy in the clinical diagnostic feature. After that, we applied single-sample Gene Set Enrichment Analysis (ssGSEA) and found a positive relation between *ZNF281* and T-cell activation in these three tumors. Then, we screened DNA methylation sites. The least absolute shrinkage and selection operator (LASS)) builds on linear regression by increasing the penalty term (lambda × absolute slope value) to reduce the overfitting of the model and improve the generalization ability of the model (Zhang et al., 2020). Statistical analysis using the LASSO technique allowed the prediction of cancer with high sensitivity and specificity (Zhang et al., 2017). Finally, we established risk prediction models based on *ZNF281*-related lncRNAs for future disease diagnosis using the LASSO technique. In conclusion, our data focused on the dual role of ZNF281 and showed that it was a potential biomarker for regeneration and might also be functional in the diagnosis and clinical prediction of multiple types of cancer.

## 2 Materials and methods

### 2.1 Preparation of decellularized adipose-derived matrix loaded with bFGF

The procedure of decellularizing adipose tissues was accomplished following our previous protocols (Tang et al., 2022). All protocols reported in this study complied with ethical regulations for work with human subjects, and the study protocol was approved by the Plastic Surgery Hospital Ethics Committee (No. ZX 201843). Briefly, human lipoaspirate from healthy women under liposuction was obtained at the Chinese Academy of Medical Sciences & Peking Union Medical College Plastic Surgery Hospital. The mixture was allowed to stand for 10–15 min; then, the upper oil and lower bloody fluids were removed. After several cycles of washing in distilled water, three freeze–thaw cycles (−80°C–37°C, 2 h each) were performed. Then, the samples were homogenized using the A11 Basic Analytical Mill (IKA, Germany) three times (28,000 rpm, 1 min each). Followed by centrifugation and removal of oil, the milky suspension was agitated in hypotonic (0.5 M NaCl, 4 h) and hypertonic solutions (1 M NaCl, 4 h). After overnight washing in distilled water, samples were soaked in 1% Triton X-100 solution, and the solution was changed three times a day for 48 h. The white floc-like precipitate was washed again in distilled water (30 min, three times), and then, 99.9% isopropanol was used for further lipid removal. After the final three repeated cycles of washing with distilled water (30 min each) and three times of agitation in 70% ethanol (30 min each) for disinfection, DAM was placed in phosphate-buffered saline (PBS) (HyClone) containing 1% penicillin–streptomycin (HyClone) at 4°C for storage.

In this study, we applied heparin cross-linking of DAM for the loading of bFGF following the previously described methods (Wissink et al., 2001; Zhang et al., 2016), the sustained releasing bFGF of which was <70% of the loaded bFGF over a period of 10 days. For loading bFGF, 250 mg wet weight DAM was mixed in 500 μL normal saline containing 2 μg/ml bFGF, and DAM was scissor-minced thoroughly. The bFGF dose has been reported as the optimal concentration for inducing adipose *in situ* regeneration (Hiraoka et al., 2006).

### 2.2 *In vivo* experiments

All animal model studies were approved in advance by the Animal Ethics Committee at the Chinese Academy of Medical Sciences & Peking Union Medical College and performed following the Animal Ethics Committee’s guidelines. Female eight-week-old C57BL/6 N mice were used for injecting the bFGF-loaded DAM mixture. As a control group, heparinized DAM mixed with PBS was used. The volume of suspension was 200 μL per site of injection (*n* ≥ 5 for each group). The animals were sacrificed at 1, 2, 3, and 12 weeks, and the samples were obtained for RNA-seq and histological analysis.

### 2.3 mRNA-Seq assay

Total RNA extracts were acquired from implanted mouse tissues of bFGF-loaded DAM and DAM at 1 week using the MagBeads Total RNA Extraction Kit (Cat#T02-096) according to the manufacturer’s instructions, and RNA integrity was checked with the RNA integrity number (RIN) by using a bioanalyzer (Agilent Technologies, US). RNA purification was performed by using the RNAClean XP kit (Cat A63987, USA) and RNase-Free DNase Set (Cat#79254, QIAGEN, Germany). After the quality control of RNA, the sequencing libraries were constructed. After library inspection using the Qubit fluorometer and Agilent 4200, the libraries were sequenced with the Illumina NovaSeq 6,000 sequencing platform, and the PE150 mode was selected. All the data were analyzed in R 3.6.4, and the R package DESeq2 was used for differential expression significance analysis. For filtrating differential expressed genes, the absolute value of log2(fold change) was selected above 2, and the *p* value was less than 0.05.

### 2.4 Histological analysis and immunofluorescence staining

After 24–48 h fixation (4% formalin), the samples retrieved from the animals were dehydrated and embedded in paraffin (Leica) for routine sectioning. The sections underwent deparaffinization and rehydration, and then, antigen retrieval was processed in a microwave in citric acid (Solarbio). After washing with PBS, slides were blocked with 5% goat serum for half an hour at 37°C, and overnight incubation with the primary antibody at 4°C was performed. Secondary immunofluorescent-tagged antibodies were used to incubate slides for 1 hour at 37°C for signal amplification. The following antibodies were used for immunofluorescence: mouse monoclonal IgG2a *κ* ZNF281 antibody (1:100, Cat. No. sc-166933, Santa Cruz), rabbit polyclonal perilipin-1 antibody (1:100, Cat. No. ab3526, Abcam), CoraLite 488-conjugated goat anti-rabbit IgG (H + L) (1:100, Cat. No. SA00013-2, Proteintech), and CoraLite 594-conjugated goat anti-mouse IgG (H + L) (1:100, Cat.No. SA00013-3, Proteintech). The nuclei were stained with DAPI (Invitrogen). Single-channel and merge images were generated in Photoshop.

### 2.5 Analysis of *ZNF281* expression in normal tissues and pan-cancer

The mRNA expression level of *ZNF281* in normal tissues was analyzed in the Human Protein Atlas (HPA) online platform (https://www.proteinatlas.org/) (Sjöstedt et al., 2020; Karlsson et al., 2021). Based on the HPA RNA-seq data and scRNA-seq data, we displayed the tissue/cell distribution of *ZNF281*, especially in normal adipose tissues. The data of RNA-seq and related clinical information were acquired from The Cancer Genome Atlas (TCGA) and the Genotype-Tissue Expression (GTEx) database using USCS Xena (http://xena.ucsc.edu/) on 16 August 2022 (Vivian et al., 2017). The data of cancer involves adrenal cortical carcinoma (ACC), bladder urothelial carcinoma (BLCA), breast-invasive carcinoma (BRCA), CESC, cholangiocarcinoma (CHOL), colon adenocarcinoma (COAD), lymphoid neoplasm diffuse large B-cell lymphoma (DLBC), esophageal carcinoma (ESCA), glioblastoma multiforme (GBM), head and neck squamous cell carcinoma (HNSC), kidney chromophobe (KICH), kidney renal clear cell carcinoma (KIRC), KIRP, acute myeloid leukemia (LAML), brain lower grade glioma (LGG), liver hepatocellular carcinoma (LIHC), lung adenocarcinoma (LUAD), lung squamous cell carcinoma (LUSC), ovarian serous cystadenocarcinoma (OV), PAAD, pheochromocytoma and paraganglioma (PCPG), prostate adenocarcinoma (PRAD), rectum adenocarcinoma (READ), skin cutaneous melanoma (SKCM), STAD, testicular germ cell tumors (TGCT), thyroid carcinoma (THCA), thymoma (THYM), uterine corpus endometrial carcinoma (UCEC), and uterine carcinosarcoma (UCS). All the data were analyzed in R 3.6.4, and the visualization was completed with the R package ggplot2. The statistical method used was the Mann–Whitney *U* test, and when *p* < 0.05, the difference was considered to reach statistical significance.

### 2.6 Survival analysis

The survival curve (also known as the Kaplan–Meier curve) can describe the survival of each group of patients. Based on the median expression of *ZNF281* or model risk scores, patients could be subdivided into the high-expression group and the low-expression group. The survminer R package was used for visualization, and the survival R package was used for the statistical analysis of survival data. OS was set as the survival outcome.

### 2.7 Receiver operating characteristic curve

The receiver operating characteristic (ROC) curve is a comprehensive index reflecting the sensitivity and specificity of continuous variables, and the composition method reflects the correlation between sensitivity and specificity. When the expression of a molecule is a trend to promote the occurrence of events, the area under the curve (AUC) of this molecule will be >0.5, and the closer the AUC approaches 1, the better the prediction performance. When the expression of a molecule is contrary to the trend of event occurrence, the molecule will be <0.5, and the closer the AUC is to 0, the more accurate the prediction performs. In brief, the point closest to the top left of the curve is the critical value with the highest sensitivity and specificity on the ROC curve. The larger the area under the curve, the higher the diagnostic accuracy. The R package pROC was used in the ROC analysis, and the ggplot2 R package was applied to visualize results (Robin et al., 2011). The expression level of *ZNF281* was used as the input in ROC analysis in 30 types of cancer. To proceed with further analysis, we selected tumors for which *ZNF281* might be recognized as a risk factor based on differential analysis, OS, ROC, and AUC; then, CESC, PAAD, and STAD were selected.

### 2.8 Single-sample Gene Set Enrichment Analysis

As an extension of Gene Set Enrichment Analysis (GSEA), single-sample Gene Set Enrichment Analysis calculates separate enrichment scores for each pairing of a sample and gene set. Gene markers for 24 immune cells were obtained from an article, and the classification and description of specific cells are shown in Bindea et al. (2013). Then, the procedures of ssGSEA were performed by the R package GSVA (version: 1.34.0) (Hänzelmann et al., 2013). Then, the relationship between the ssGSEA scores of 24 immune cells and *ZNF281* expression in CESC, PAAD, and STAD was calculated by Spearman correlation analysis.

### 2.9 *ZNF281*-associated DNA methylation sites

The correlation between the beta value of methylation sites within 5000bp upstream and downstream of the transcription start site (TSS) and the expression level of *ZNF281* was calculated. Spearman correlation analysis was included. The stat R package was used. DNA methylation sites with *p* value less than 0.05 and Spearman correlation coefficient greater than 0.3 were selected.

### 2.10 Screening *ZNF28*1-related genes

In CESC, PAAD, and STAD, we investigated the correlation between other genes and *ZNF281* expression *via* Spearman and Pearson correlation analyses. To find genes with statistical significance, *P* (Spearman) and *p* (Pearson) less than 0.05 were selected as the basic screening rule. Genes with correlation coefficient (Cor) more than 0.55 or less than −0.55 for both Spearman and Pearson analyses in CESC and STAD were screened. For PAAD, the absolute value of Cor (Spearman) was filtered over 0.7 and the absolute value of Cor (Pearson) was filtered over 0.6. This procedure was achieved *via* the stat R package.

### 2.11 Least absolute shrinkage and selection operator

The expression of *ZNF281*-related genes was selected as the input in LASSO. In the process of ten-fold cross validation, the seed number was set as 2021. The screening threshold of the model coefficients was selected as lambda. min. According to the selected genes by LASSO, we built the risk models in CESC, PAAD, and STAD. Also, the LASSO coefficients were used to calculate the risk scores with the expression of genes in models. The glmnet and survival R packages were used.

### 2.12 Time-dependent receiver operating characteristic curve

Time-dependent receiver operating characteristic (tdROC) curve analysis was mainly used to analyze the predictive efficacy of one continuous variable in predicting outcomes related to time. The tdROC was used to complete the analysis, and ggplot2 was used to visualize the results. Also, the tdROC results of the expression of *ZNF281* at 1, 3, and 5 years were calculated.

## 3 Results

### 3.1 New adipose formation and concomitant *zfp281* expression upregulated at an early stage

**FIGURE 1:**
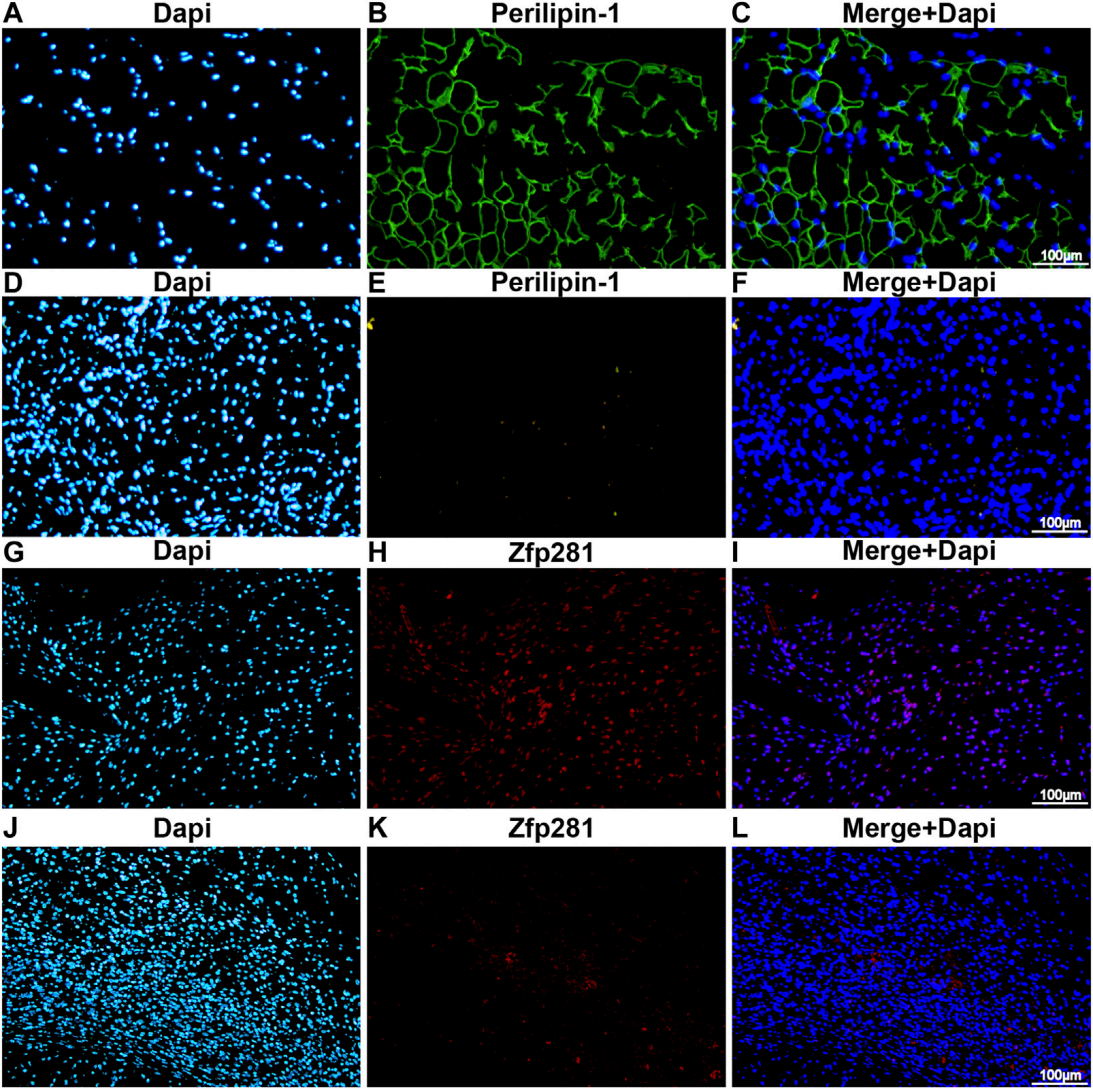
Zfp281 is upregulated at an early stage in adipose *de novo* regeneration tissues. **(A–F)** Immunofluorescence staining of perilipin-1 in bFGF-loaded DAM **(A–C)** and DAM-injected mouse tissuees **(D–F)** at 12 weeks; **(G–L)** immunofluorescence staining of zfp281 in bFGF-loaded DAM **(G–I)** and DAM-injected mouse tissues **(J–L)** at 1 week. Perilippin-1 is labeled in green. Zfp281 is Labeled in red. Nuclei are labeled with DAPI in blue.

The differential gene expression of RNA-seq is displayed in Supplementary Figure S1A, and a significant fold difference in *zfp281* is shown between the two groups. Compared to the control group (Figures 1D–F), samples derived from bFGF-loaded DAM (Figures 1A–C **)** showed a significant adipose regeneration phenomenon. Moreover, zfp281 expression in the early stage (1 week) was validated higher in the bFGF-loaded DAM group (Figures 1G–L).

### 3.2 Gene expression in normal tissues and pan-cancer

**FIGURE 2:**
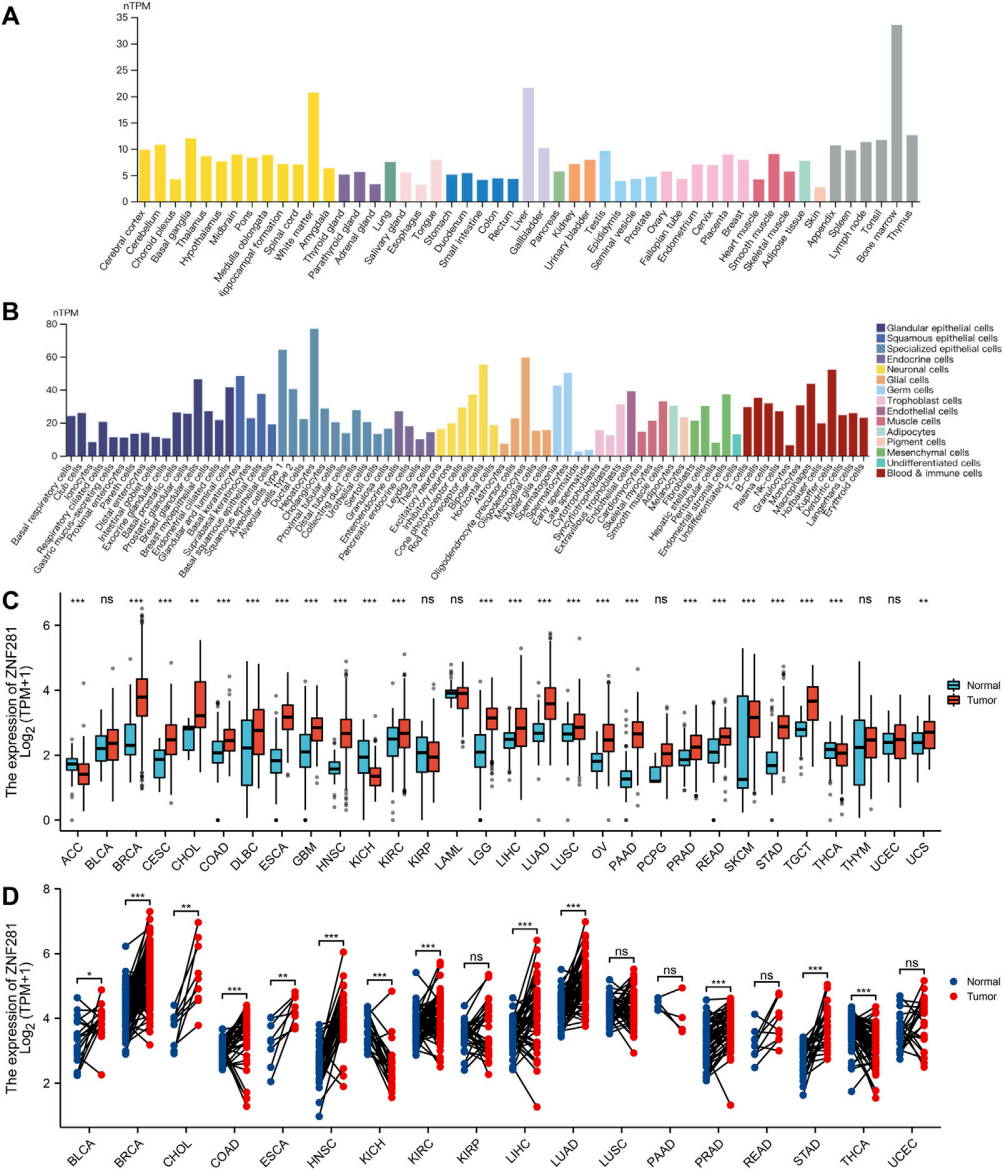
Expression levels of *ZNF281* were higher in tumors and normal tissues. **(A)** *ZNF281* expression in normal tissues, **(B)** *ZNF281* expression in single-cell types, **(C)** *ZNF281* expression was upregulated in most types of cancers than normal tissues, combining the data of TCGA and the GTEx database, and **(D)** *ZNF281* expression in TCGA tumors and adjacent normal tissues (^*^ *p* < 0.05, ^**^ *p* < 0.01, and ^***^ *p* < 0.001).

As shown in Figures 2A, B, we found *ZNF281* was highly expressed in the bone marrow, followed by the liver and white matter. Moreover, the cell type that expressed the highest level of *ZNF281* is hepatocytes. In adipose tissues (Supplementary Figure S2B), a cluster of macrophages express the highest amount of *ZNF281* mRNA. We compared *ZNF281* expression levels across 30 types of tumors and relevant normal tissues. In most types of cancers, *ZNF281* was expressed significantly higher in tumors (Figures 2C, D). However, *ZNF281* was less expressed in three types of tumors compared to corresponding normal tissues, including ACC, KICH, and THCA. By integrating data from the GTEx database, we compared the *ZNF281* expression levels in cancer and their normal adjacent tissues, and *ZNF281* was significantly expressed higher in 11 cancer types, including BLCA, BRCA, CHOL, COAD, ESCA, HNSC, KIRC, LIHC, LUAD, PRAD, and STAD. Meanwhile, in KICH and THCA, *ZNF281* was downregulated in cancer.

### 3.3 Higher expression of *ZNF281* in a variety of tumors suggests a poorer prognosis

**FIGURE 3:**
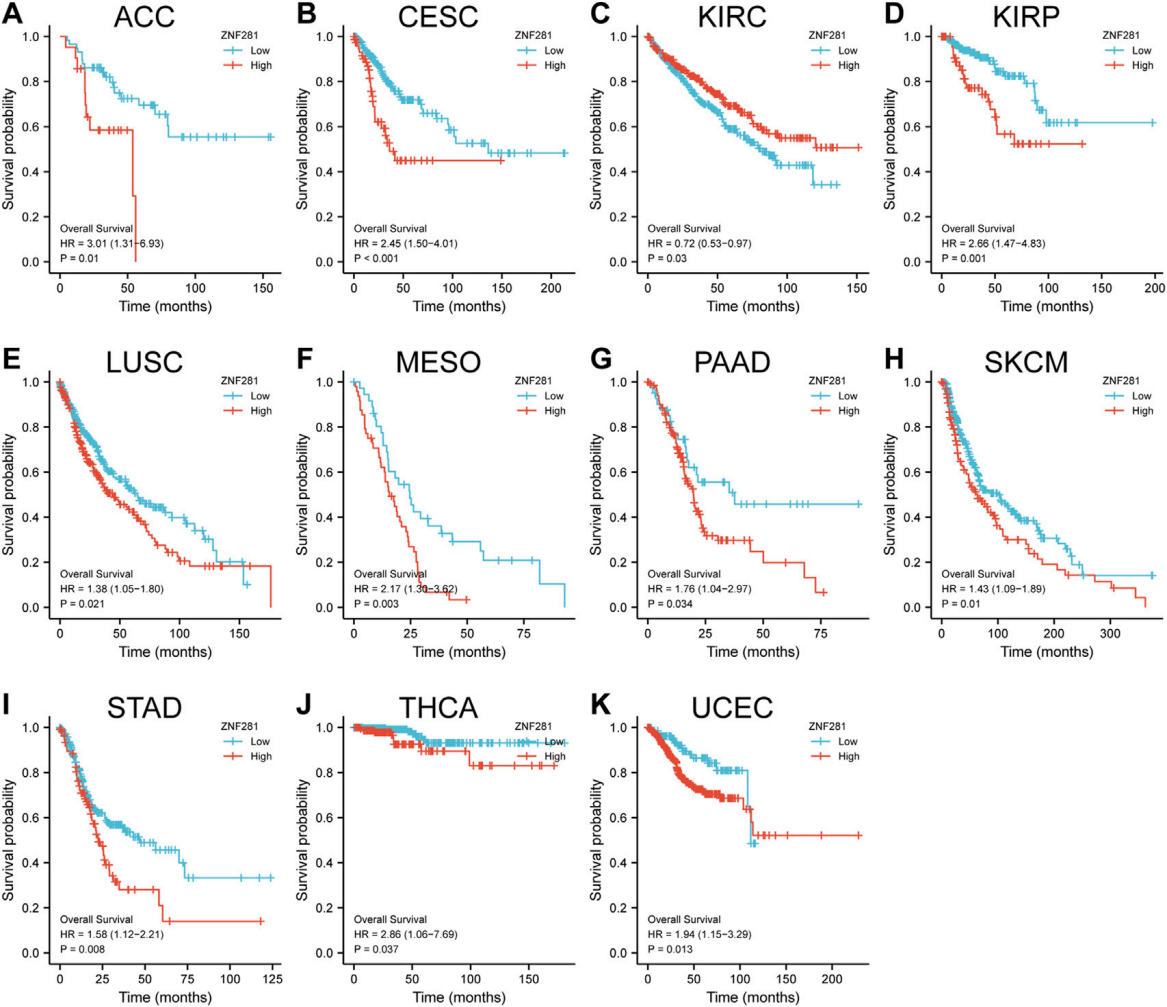
Correlations between the *ZNF281* expression and prognosis (OS) of patients in different cancers. **(A)** ACC, **(B)** CESC, **(C)** KIRC, **(D)** KIRP, **(E)** LUSC, **(F)** MESO, **(G)** PAAD, **(H)** SKCM, **(I)** STAD, **(J)** THCA, and **(K)** UCEC.

According to the results of survival analyses (Figure 3), patients with *ZNF281* expression above the median value had worse OS in ACC (*p* = 0.01, HR = 3.01), CESC (*p* < 0.001, HR = 2.45), KIRP (*p* = 0.001, HR = 2.66), LUSC (*p* = 0.021, HR = 1.38), MESO (*p* = 0.003, HR = 2.17), PAAD (*p* = 0.034, HR = 1.76), SKCM (*p* = 0.01, HR = 1.43), STAD (*p* = 0.008, HR = 1.58), THCA (*p* = 0.037, HR = 2.86), and UCEC (*p* = 0.013, HR = 1.94). However, patients with lower *ZNF281* expression levels based on the median value showed the worst prognosis in KIRC (*p* = 0.03, HR = 0.72). However, in the other types of cancer, *ZNF281* was not related to prognosis, and the results are shown in Supplementary Figure S2.

### 3.4 *ZNF281* has good diagnostic efficacy in CESC, STAD, and PAAD

**FIGURE 4:**
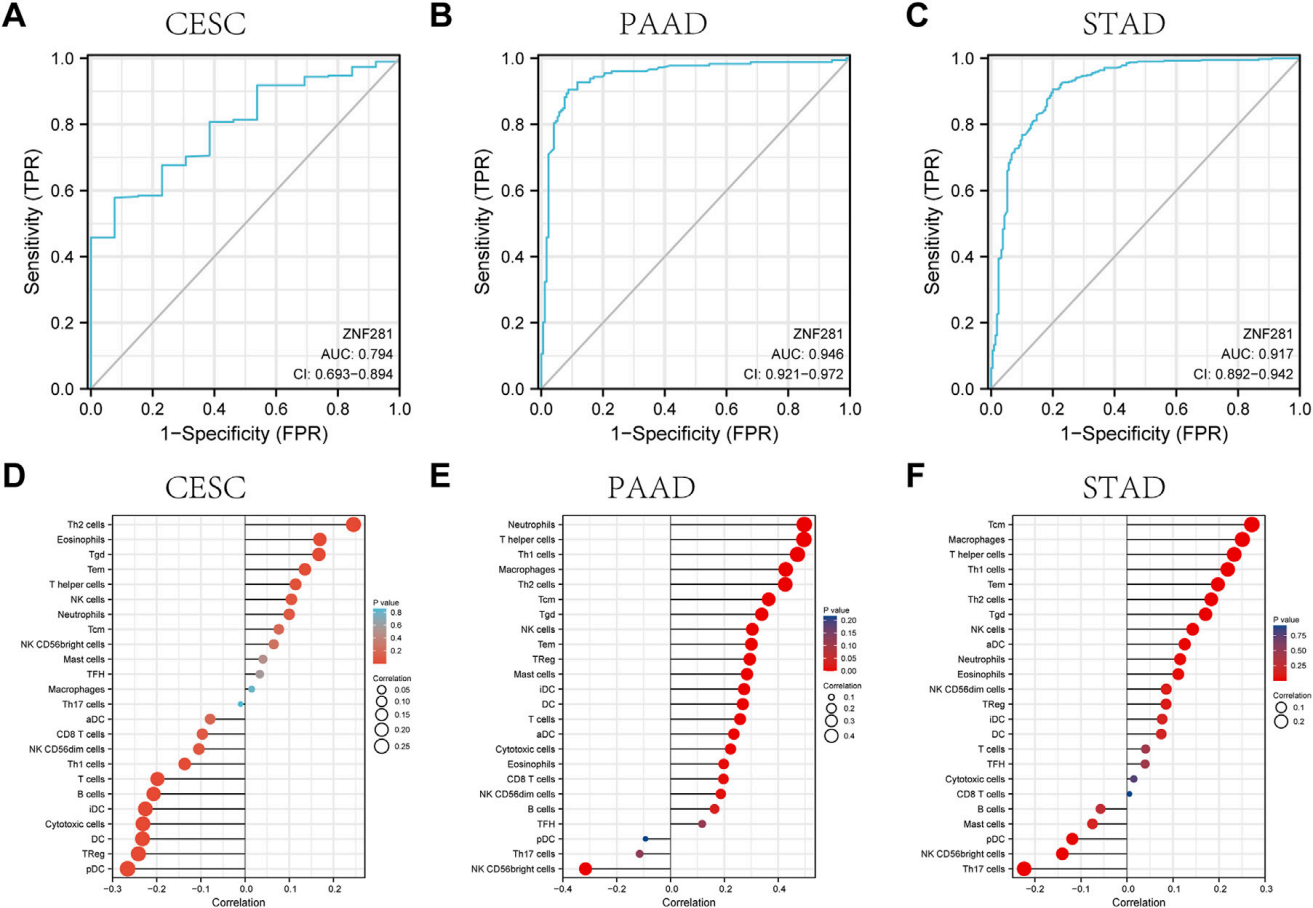
ROC curve and the relationship with immune cell infiltration for *ZNF281* in CESC, PAAD, and STAD. **(A)** ROC curve for *ZNF281* in CESC, **(B)** ROC curve for *ZNF281* in PAAD, and **(C)** ROC curve for ZNF281 in STAD. **(D)** Immune infiltration analysis for *ZNF281* in CESC, **(E)** immune infiltration analysis for *ZNF281* in PAAD, and **(F)** immune infiltration analysis for *ZNF281* in STAD.

According to ROC analysis, *ZNF281* showed a good diagnostic ability in differentiating tumors from benign tissues in CESC (Figure 4A, AUC = 0.794), STAD (Figure 4B, AUC = 0.946), and PAAD (Figure 4C, AUC = 0.917). The ROC analysis in the other types of tumors is shown in Supplementary Figure S3.

### 3.5 ZNF281 is mainly related to the activation of the T-cell family

As shown in Figure 4D, *ZNF281* was positively correlated to the levels of Th2 cells, eosinophils, T gamma delta cells, T effector memory cells, and T helper cells in CESC. Also, according to Figure 4E, more expressions of *ZNF281* indicated more levels of neutrophils, T helper cells, Th1 cells, macrophages, and Th2 cells in PAAD. In STAD (Figure 4F), the levels of *ZNF281* were associated with the content of T central memory cells, macrophages, T helper cells, Th1 cells, and T effector memory cells.

### 3.6 Two DNA methylation sites in the TSS region of *ZNF281* may regulate its transcription activity in PAAD

**FIGURE 5:**
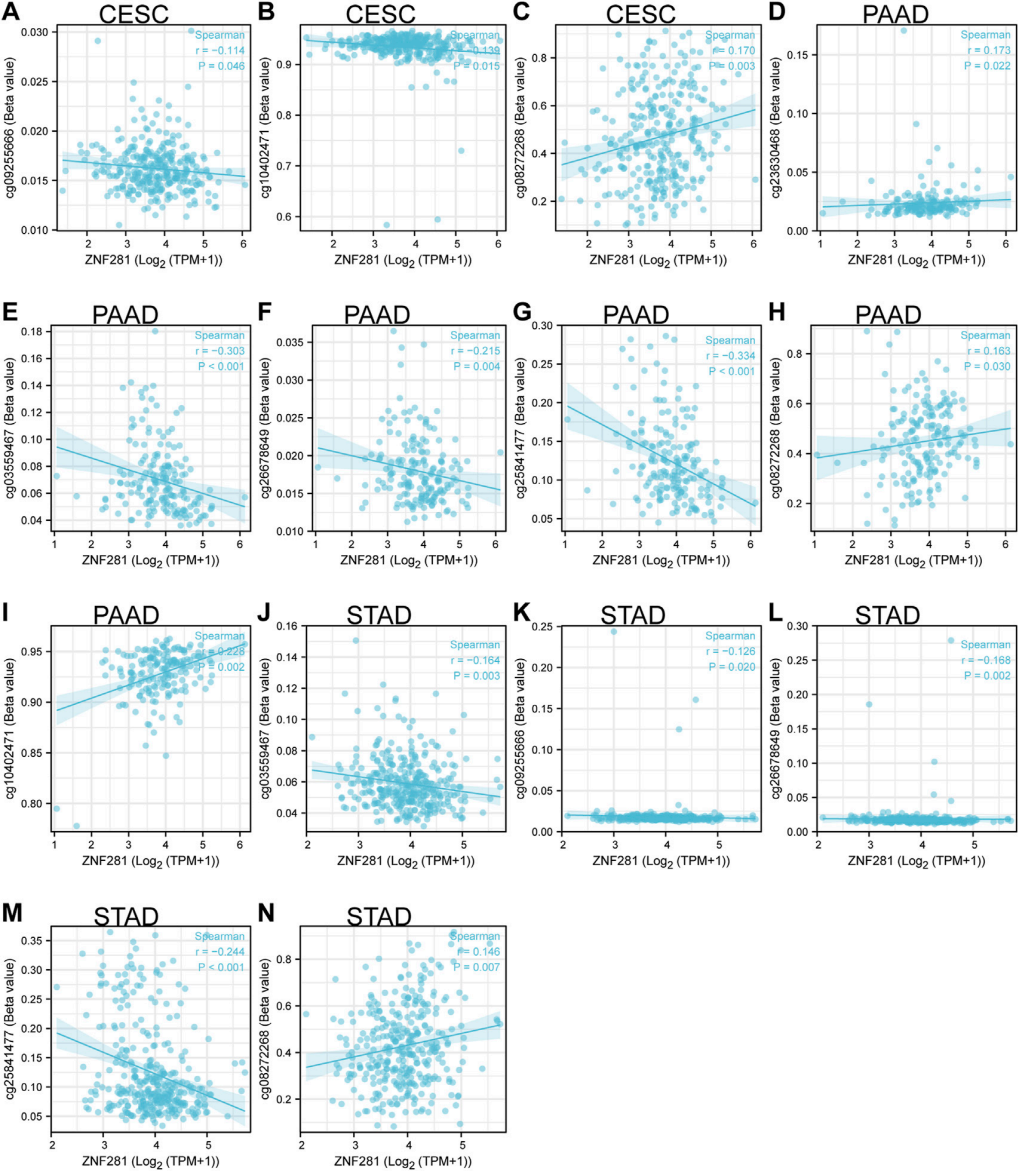
*ZNF281*-associated DNA methylation sites in CESC, PAAD, and STAD. **(A–C)** Methylation sites in the TSS region of *ZNF281* in CESC, **(D–I)** methylation sites in the TSS region of *ZNF281* in CESC, and **(J–N)** methylation sites in the TSS region of *ZNF281* in CESC.

Methylation is mainly carried out through DNA methyltransferase to add methyl groups to DNA and to affect the DNA transcription process without changing the DNA sequence. Based on the screening conditions of *p* < 0.05 and Spearman correlation coefficient >0.3, two DNA methylation sites (cg03559467: correlation coefficient = −0.303, *p* < 0.001 and cg25841477: correlation coefficient = −0.334, *p* < 0.001) might be related to the transcription of *ZNF281* in PAAD (Figures 5E, G). However, the transcriptional regulation of *ZNF281* did not seem to be related to DNA methylation in STAD and CESC (Figure 5).

### 3.7 Three lncRNA prognostic models based on ZNF281-correlated genes were constructed in CESC, PAAD, and STAD

**FIGURE 6:**
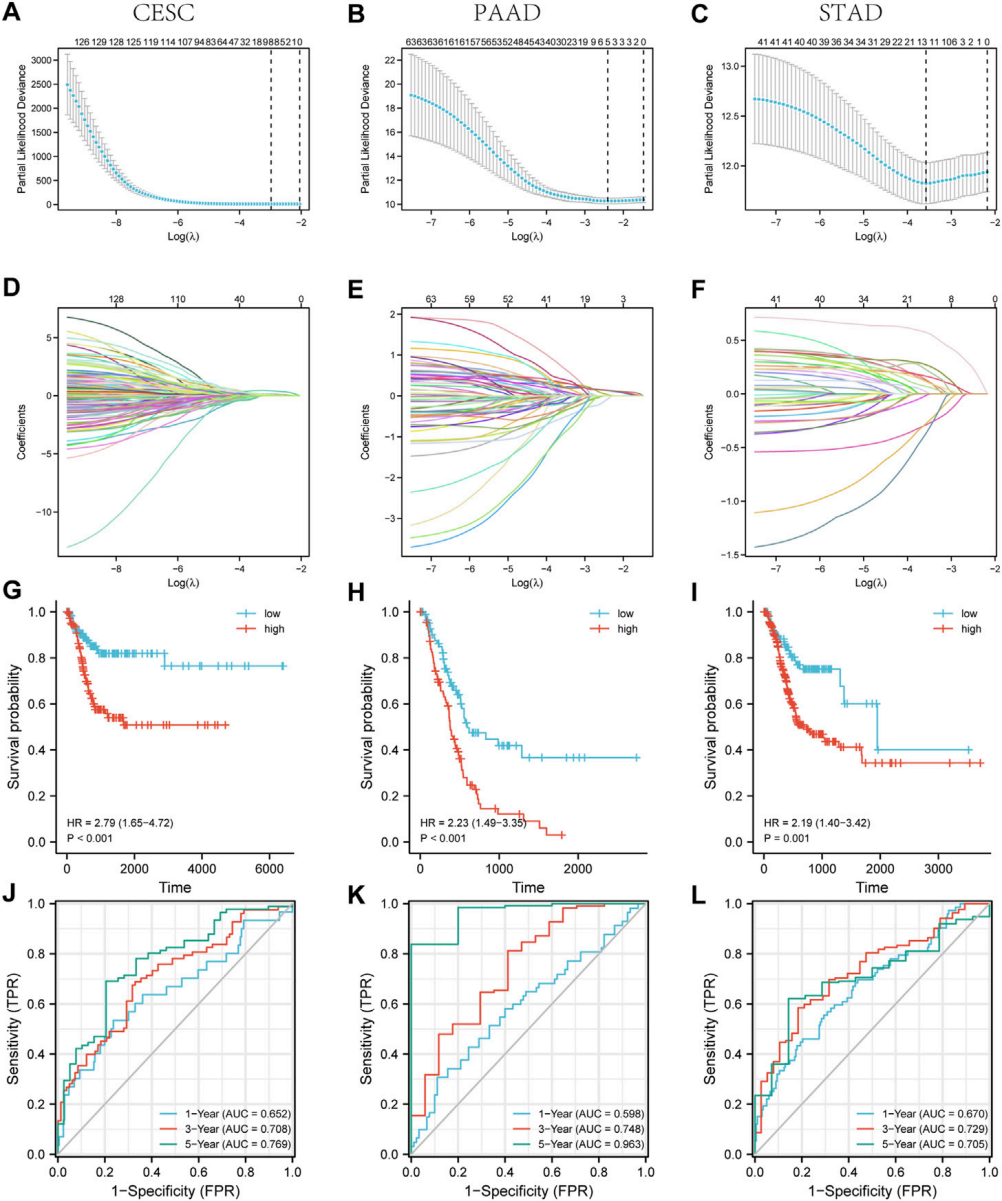
Three lncRNA prognostic models associated with *ZNF281* showed good accuracy in predicting the prognosis of patients in CESC, PAAD, and STAD. **(A–C)** Cross validation based on C-index to determine the best choice of genes in the prediction models. **(D–F)** Genes in the different choices of models and their corresponding coefficients based on different lambda values. **(G–I)** According to prediction models, the relationship between the survival outcome and risk levels of patients. **(J–L)** The tdROC curve of 1, 3, and 5 years verified the efficacy of prediction models. **(A,D,G,J)** CESC, **(B,E,H,K)** PAAD, and **(C,F,I,L)** STAD.

We screened 129 lncRNA coding genes in CESC (Supplementary Table S1), 63 lncRNA coding genes in PAAD (Supplementary Table S2), and 41 lncRNA coding genes in STAD (Supplementary Table S3) associated with the *ZNF281* level. The expression of these selected lncRNA coding genes was used as the input to train the prognostic models in CESC, PAAD, and STAD. As shown in Figures 6A–C, each point in the figure represented the mean value of likelihood deviation corresponding to each lambda in the process of cross validation, and the error line represents the corresponding error situation. In general, a smaller likelihood deviation value corresponds to a better model, which corresponds to the lambda. min value. According to the lambda. min value, genes in the final models were selected and their coefficients are shown in Figures 6D–F. The risk score can be calculated as follows: CESC: risk score = BOLA3-AS1 × 0.048 + AC139887.2 × 0.024 + NADK2-AS1 × 0.081 + NKILA × 0.130 + LINC01719 × 0.417 + AC022784.5 × 0.068 + AP001094.2 × 0.103 + AL365436.2 × 0.083; PAAD: risk score = AC099850.3 × 0.139 + AP003119.3 × 0.133 + AC112721.2 × 0.129 + AC073046.1 × 0.032—HCG18 × 0.073; and STAD: risk score = ERICD × 0.573 + AC105036.3 × 0.272 + ZNF8-ERVK3-1 × 0.226 + NKILA × 0.166 + PTOV1-AS1×0.132 + AC005332.6 × 0.124 + AP000759.1 × 0.114 + STARD4-AS1 × 0.070 + LINC00205 × 0.019—AC092171.2 × 0.013—AC107068.1 × 0.276—AL355574 × 0.288—LINC00630 × 0.367. The higher risk score is related to a worse prognosis. As shown in Figures 6G–I, patients with higher risk scores presented worse OS in CESC (*p* < 0.001, HR = 2.79), PAAD (*p* < 0.001, HR = 2.23), and STAD (*p* < 0.001, HR = 2.19). Furthermore, these three lncRNA prognostic models for CESC, PAAD, and STAD exhibited good predictive ability for PFI at 1, 3, and 5 years (Figures 6J–L).

## 4 Discussion

We hypothesized that ZNF281 (zfp281 in *Mus musculus*) would be a potential biomarker in some forms of regeneration and tumorigenesis, which share similar molecular pathways. We first detected the upregulation of *zfp281* in *de novo* adipogenesis through RNA-seq and then validated the histological expression in the early stage of the adipose regeneration model. Through literature review, we found that ZNF281 was related to the tumor development and progression mechanism, such as EMT (Hahn et al., 2013; Pierdomenico et al., 2018; Sadłecki et al., 2019; Xue et al., 2019). Considering the heterogeneity and complexity of tumors, we analyzed the *ZNF28*1 expression in pan-cancer and related normal tissues using RNA-seq data from TCGA and GTEx and found the upregulation of *ZNF281* in most types of cancer. Moreover, the *ZNF281* expression level was related to worse prognosis in 10 types of cancer. However, in KIRC, higher expression of *ZNF281* was expected to have survival benefits given the OS analysis. Further investigation indicated *ZNF281* had good prognostic value in CESC, PAAD, and STAD. Also, certain types of T-cell activation were positively correlated to the *ZNF281* expression. In addition, we explored two DNA methylation sites in the TSS region of *ZNF281* that may regulate its transcription activity in PAAD and could be targeted when treating PAAD. Finally, we established clinical prognostic models based on *ZNF281*-related lncRNA in CESC, PAAD, and STAD.

ZNF281 has also shown its role in regeneration regulation. Zhou et al. (2017) found ZNF281 enhances cardiac reprogramming and upregulation of ZNF281 significantly activated genes related to myogenesis, muscle contraction, and heart processes. As a strong activator in cardiac reprogramming, ZNF281 promotes cardiac regeneration by working on GATA4, which represents an important cardiogenic transcription factor (Lalit et al., 2017; Stone et al., 2019), and works against inflammatory reactions, which inhibits cardiac reprogramming. Here, we hypothesized three possible explanations for elevated zfp281 at the early stages of adipose regeneration. First, that might be the result of environmental response to regeneration signals, which promotes cell proliferation and migration. A mesenchymal-like program as negative feedback is exerted by the environment to keep homeostasis, and then, the expression of zfp281 might be upregulated (Kciuk et al., 2022; Nobre et al., 2022). Second, ZNF281 might promote regeneration by activating EMT-related pathways (Hahn et al., 2013). EMT is divided into three different subtypes: type-1 EMT, type-2 EMT, and type-3 EMT. Type-2 EMT is correlated to the wound healing process, tissue regeneration, and organ fibrosis (Oh et al., 2018; Aharonov et al., 2020; Klatt Shaw et al., 2021; Marconi et al., 2021). Activation of EMT has been linked to the acquisition of both normal and neoplastic stem cells, which might lead to regeneration and carcinogenesis, respectively (Lambert and Weinberg, 2021). Finally, ZNF281 has been recognized as a direct participant in DNA damage response and repair (DRR), and the upregulation of DDR-related molecules reduces tumor immunogenicity and then causes neoplastic cells to escape from the immune system (Nicolai et al., 2020a). A similar mechanism may also exist in regenerating cells.

ZNF281 inhibits the differentiation of neurons, and higher expression was proven related to a worse prognosis in neuroblastoma (Pieraccioli et al., 2018). Moreover, ZNF281 is mainly expressed in poorly differentiated cells and tissues, and accompanying differentiating process, the expression level of ZNF281 was downregulated. Nicolai et al. (2020b) proposed that ZNF281 directly inhibits muscle differentiation promoted by microRNA-1. Furthermore, they validated the higher expression of ZNF281 in certain types of soft-tissue sarcoma, such as rhabdomyosarcoma and leiomyosarcoma tumors. In our research, we found that ZNF281 was expressed higher in most tumors than in the corresponding normal tissues. Moreover, reduced survival expectations were observed in high ZNF281 cancer. These findings can also be explained by EMT and DDR mechanisms, which are related to metastasis and immune escape of tumors. Beyond that, we validated the good prognostic value of ZNF281 in CESC, STAD, and PAAD and finally established risk prediction models based on ZNF281-related molecules for future clinical diagnosis and treatment.

Sharing common molecular pathways, more and more evidence indicates that regeneration and carcinogenesis are related processes, although accompanying diverse outcomes are life and death (Ewerbeck et al., 1985; Sarig and Tzahor, 2017). In this context, the activity of ZNF281 might be recognized as a potential biomarker for regeneration and cancer. However, there are still some limitations. First, the risk model does not apply an external database to verify its effectiveness. Second, extensive further work is required to understand the mechanism that how ZNF281 is regulated in the specific regeneration-promoted environment and how ZNF281, in turn, regulates regeneration. Further interactions between regeneration and tumorigenesis should also be uncovered in future scientific research. Moreover, in this study, we utilized shallow machine learning to build the risk model other than deep learning, which is more flexible.

## Data Availability

The datasets presented in this study can be found in online repositories. The names of the repository/repositories and accession number(s) can be found in the article/Supplementary Material.
