# Supplementary material for: Multi-functional gene ZNF281 identified as a molecular biomarker in soft tissue regeneration and pan-cancer progression
Source: Front Genet. 2023 Jan 5;13:1082654. doi: 10.3389/fgene.2022.1082654 (PMC9849369; doi:10.3389/fgene.2022.1082654)
Supplement: Supplementary file 1 [file Table1.DOCX]

Supplementary Material

# Supplementary Figures Supplementary Figure 1
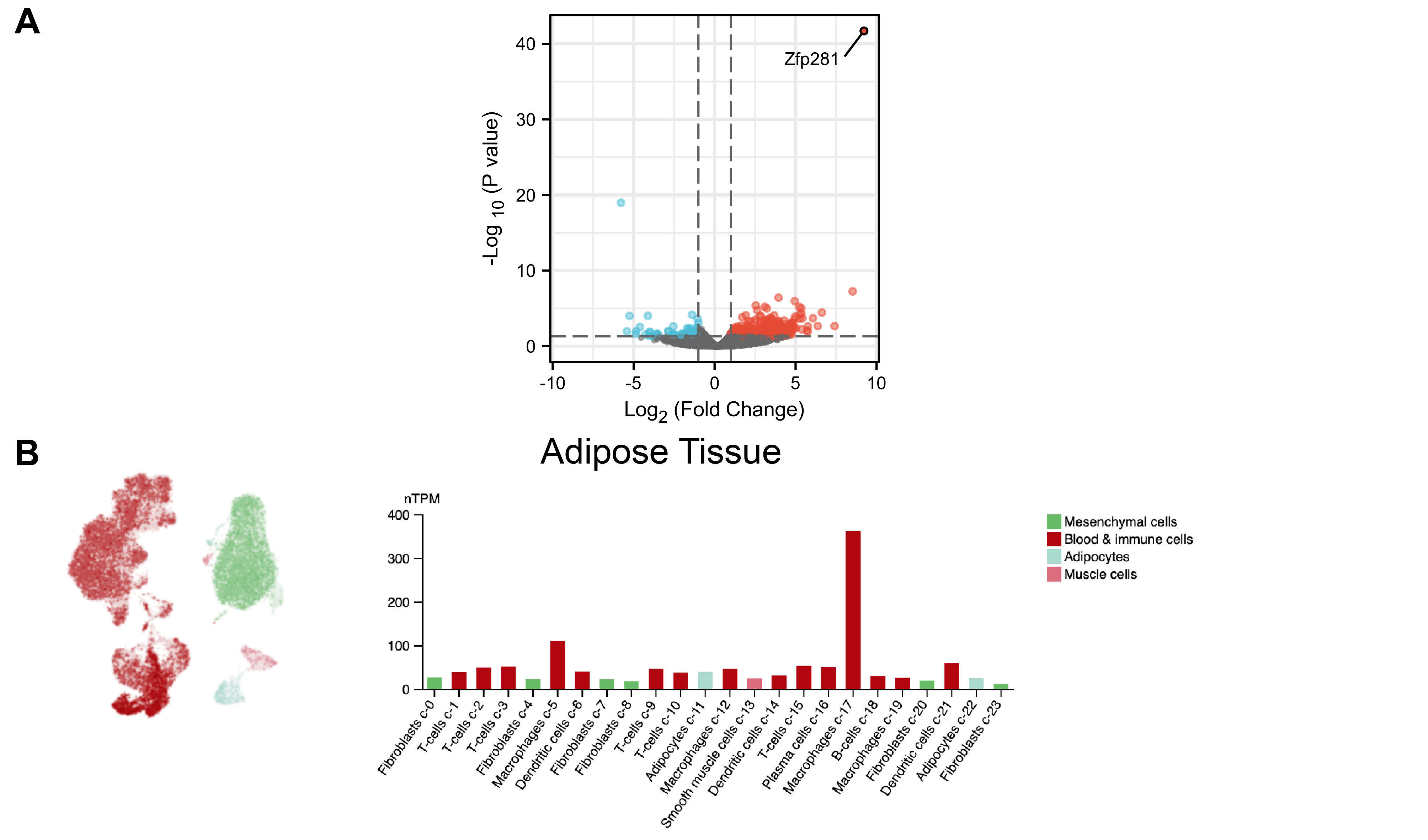


**Supplementary Figure 1.** Higher *zfp281* expressed in the adipose regeneration model and scRNA of *ZFP281* expression in human adipose tissue. **(A)** Volcano plots of differential genes of mouse RNA-seq; **(B)** Single cell expression of *ZNF281* expression in adipose tissue.

# Supplementary Figures Supplementary Figure 2


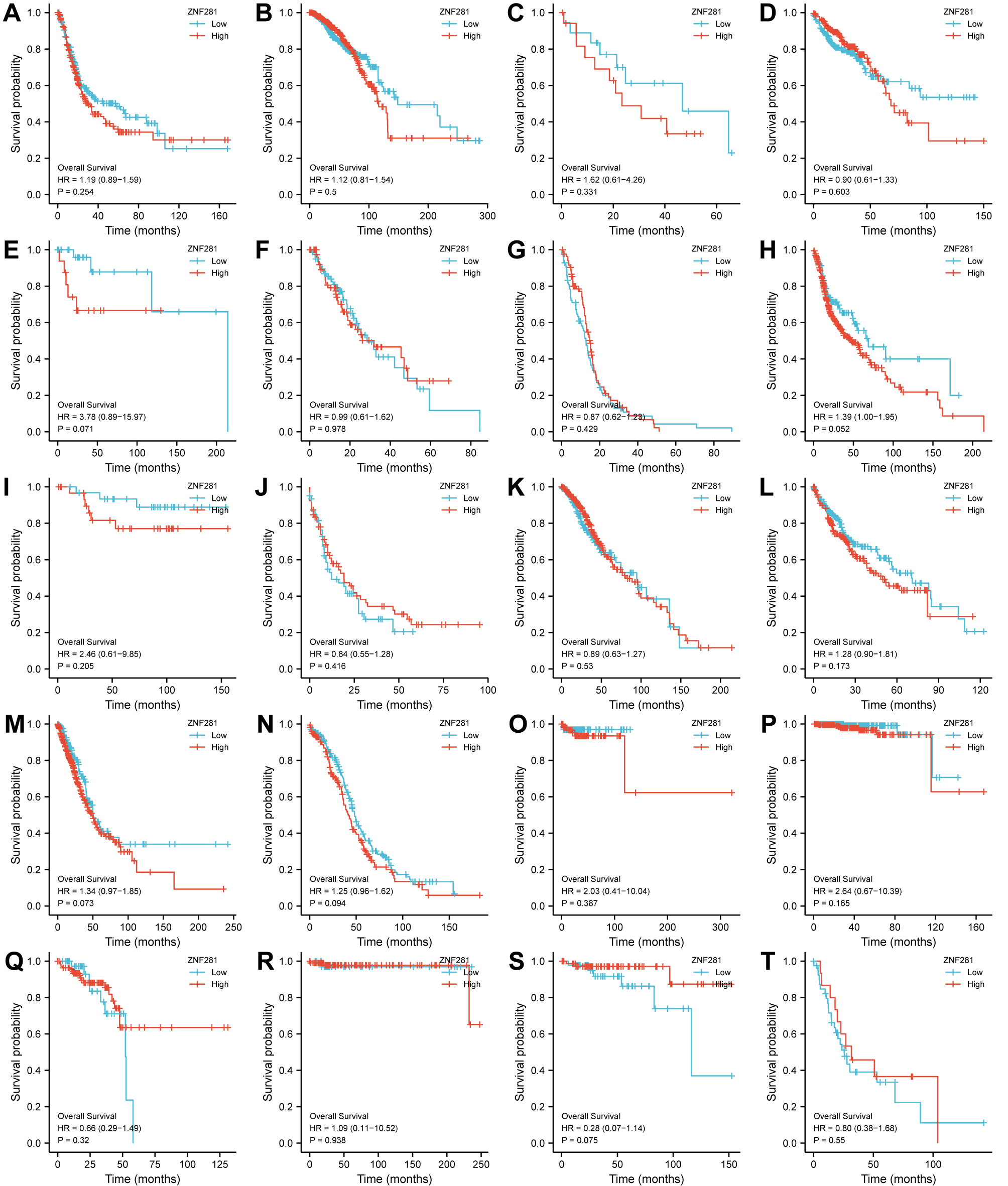


**Supplementary Figure 2.** Correlations between ZNF281 expression and the prognosis (OS) of patients in different cancers. **(A)** BLCA; **(B)** BRCA; **(C)** CHOL; **(D)** COAD; **(E)** DLBC; **(F)** ESCA; **(G)** GBM; **(H)** HNSC; **(I)** KICH; **(J)** LAML; **(K)** LGG; **(L)** LIHC; **(M)** LUAD; **(N)** OV; **(O)** PCPG; **(P)** PRAD; **(Q)** READ; **(R)** TGCT; **(S)** THYM; **(T)** UCS

**Supplementary Figure 3**


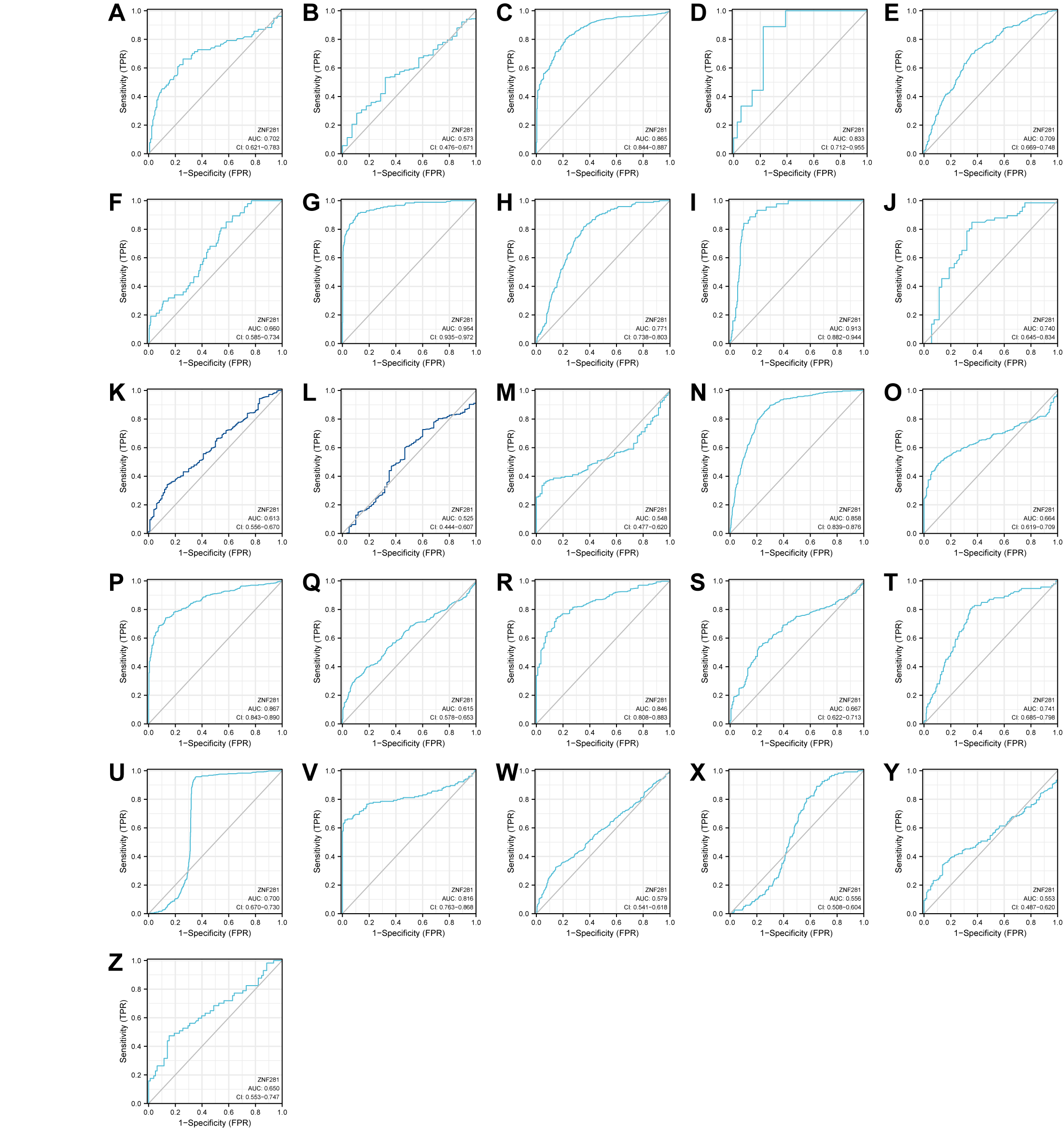


**Supplementary Figure 3.** Receiver operating characteristic (ROC) curve for ZNF281 in different types of cancers. **(A)** ACC; **(B)** BLCA; **(C)** BRCA; **(D)** CHOL; **(E)** COAD; **(F)** DLBC; **(G)** ESCA; **(H)** GBM; **(I)** HNSC; **(J)** KICH; **(K)** KIRC; **(L)** KIRP; **(M)** LAML; **(N)** LGG; **(O)** LIHC; **(P)** LUAD; **(Q)** LUSC; **(R)** OV; **(S)** PRAD; **(T)** READ; **(U)** SKCM; **(V)** TGCT; **(W)** THCA; **(X)** THYM; **(Y)** UCEC; **(Z)** UCS.

# Supplementary Tables

**Supplementary Table 1: Selected 129 lncRNAs related to expression of ZNF281 in CESC**

| **Supplementary Table 1: Selected 129 lncRNAs related to expression of ZNF281 in CESC** | | | | | | |
| --- | --- | --- | --- | --- | --- | --- |
| gene_name | gene_id | gene_biotype | cor_pearson | p_pearson | cor_spearman | p_spearman |
| AP000941.1 | ENSG00000255176 | lncRNA | 0.55405478 | 2.98E-11 | 0.58740547 | 9.226E-13 |
| AC016722.2 | ENSG00000228925 | lncRNA | 0.61743843 | 2.82E-14 | 0.65248824 | 2.927E-16 |
| BOLA3-AS1 | ENSG00000225439 | lncRNA | 0.57133401 | 5.17E-12 | 0.59205995 | 0 |
| AP001001.1 | ENSG00000254433 | lncRNA | 0.5682599 | 7.11E-12 | 0.60957971 | 7.289E-14 |
| AC105389.2 | ENSG00000249216 | lncRNA | 0.57026914 | 5.77E-12 | 0.57653166 | 2.99E-12 |
| AL049840.4 | ENSG00000269910 | lncRNA | 0.6661436 | 4.18E-17 | 0.6180818 | 0 |
| AP001020.3 | ENSG00000266171 | lncRNA | 0.55878182 | 1.86E-11 | 0.58642633 | 1.028E-12 |
| AL139099.2 | ENSG00000258450 | lncRNA | 0.61512662 | 3.74E-14 | 0.62972526 | 6.08E-15 |
| AL354989.1 | ENSG00000228352 | lncRNA | 0.59455845 | 4.16E-13 | 0.59782234 | 2.869E-13 |
| AL365203.2 | ENSG00000273038 | lncRNA | 0.66114225 | 8.62E-17 | 0.65833667 | 0 |
| AC078778.1 | ENSG00000258344 | lncRNA | 0.58051253 | 1.95E-12 | 0.60218447 | 1.737E-13 |
| LINC00641 | ENSG00000258441 | lncRNA | 0.64267803 | 1.12E-15 | 0.61352233 | 0 |
| AC118344.1 | ENSG00000205041 | lncRNA | 0.57289949 | 4.39E-12 | 0.63812561 | 2.044E-15 |
| OTUD6B-AS1 | ENSG00000253738 | lncRNA | 0.59584676 | 3.59E-13 | 0.55195341 | 0 |
| RAB11B-AS1 | ENSG00000269386 | lncRNA | -0.5940615 | 4.4E-13 | -0.5858624 | 0 |
| AC018653.3 | ENSG00000256967 | lncRNA | 0.58809033 | 8.56E-13 | 0.57662741 | 0 |
| AP001793.1 | ENSG00000266708 | lncRNA | 0.55570252 | 2.53E-11 | 0.55279556 | 3.368E-11 |
| AC073130.2 | ENSG00000243243 | lncRNA | 0.58946896 | 7.34E-13 | 0.57343852 | 4.145E-12 |
| AL139035.1 | ENSG00000280710 | lncRNA | 0.59414278 | 4.36E-13 | 0.61957611 | 2.173E-14 |
| AC024909.1 | ENSG00000274021 | lncRNA | 0.56167599 | 1.39E-11 | 0.65960501 | 1.074E-16 |
| XIST | ENSG00000229807 | lncRNA | 0.60852683 | 8.26E-14 | 0.58710064 | 0 |
| AC138028.2 | ENSG00000224888 | lncRNA | 0.57932399 | 2.22E-12 | 0.61289074 | 4.905E-14 |
| AC000120.2 | ENSG00000243107 | lncRNA | 0.57509841 | 3.48E-12 | 0.5895262 | 7.298E-13 |
| AC005288.1 | ENSG00000266469 | lncRNA | 0.66127276 | 8.46E-17 | 0.65627942 | 0 |
| AC125494.1 | ENSG00000219410 | lncRNA | 0.62726441 | 8.32E-15 | 0.63382948 | 3.584E-15 |
| AC108449.2 | ENSG00000259366 | lncRNA | 0.66575077 | 4.42E-17 | 0.63873572 | 1.886E-15 |
| NORAD | ENSG00000260032 | lncRNA | 0.59423558 | 4.31E-13 | 0.57876849 | 0 |
| AL450326.1 | ENSG00000230555 | lncRNA | 0.59869938 | 2.6E-13 | 0.57605072 | 3.147E-12 |
| AC129510.1 | ENSG00000265678 | lncRNA | 0.60312884 | 1.56E-13 | 0.57739448 | 2.728E-12 |
| AL118506.1 | ENSG00000268858 | lncRNA | 0.61992636 | 2.08E-14 | 0.61899111 | 0 |
| AC092574.2 | ENSG00000281016 | lncRNA | 0.57409573 | 3.87E-12 | 0.63308705 | 3.946E-15 |
| Z99572.1 | ENSG00000213062 | lncRNA | 0.64528815 | 7.86E-16 | 0.68110306 | 4.383E-18 |
| AL731571.1 | ENSG00000273599 | lncRNA | 0.61838176 | 2.52E-14 | 0.61099431 | 0 |
| AC093752.3 | ENSG00000260091 | lncRNA | 0.55092071 | 4.05E-11 | 0.55007386 | 4.393E-11 |
| FGD5-AS1 | ENSG00000225733 | lncRNA | 0.61606607 | 3.34E-14 | 0.58900311 | 0 |
| AP001350.1 | ENSG00000269570 | lncRNA | 0.5746225 | 3.66E-12 | 0.58408341 | 1.328E-12 |
| AC104561.1 | ENSG00000253390 | lncRNA | 0.55876597 | 1.86E-11 | 0.56282116 | 1.24E-11 |
| ANKRD10-IT1 | ENSG00000229152 | lncRNA | 0.65697144 | 1.56E-16 | 0.62108705 | 0 |
| AC012313.5 | ENSG00000268912 | lncRNA | 0.55666452 | 2.3E-11 | 0.58677174 | 0 |
| AL359922.2 | ENSG00000265194 | lncRNA | 0.59681193 | 3.22E-13 | 0.62891066 | 6.746E-15 |
| AC020907.4 | ENSG00000271032 | lncRNA | 0.59717083 | 3.09E-13 | 0.61245352 | 5.169E-14 |
| AC092614.1 | ENSG00000227542 | lncRNA | 0.6043562 | 1.35E-13 | 0.60221725 | 1.73E-13 |
| AC007878.1 | ENSG00000281195 | lncRNA | 0.56584696 | 9.11E-12 | 0.55704101 | 2.215E-11 |
| EBLN3P | ENSG00000281649 | lncRNA | 0.64872213 | 4.92E-16 | 0.63232771 | 0 |
| AC135050.5 | ENSG00000262766 | lncRNA | 0.60347822 | 1.49E-13 | 0.55123459 | 3.924E-11 |
| AL731577.2 | ENSG00000249456 | lncRNA | 0.63243855 | 4.29E-15 | 0.61015223 | 6.808E-14 |
| AC099850.3 | ENSG00000265415 | lncRNA | 0.70264211 | 1.34E-19 | 0.68708646 | 0 |
| AL035448.1 | ENSG00000236526 | lncRNA | 0.615384 | 3.63E-14 | 0.70287522 | 1.284E-19 |
| AC010834.3 | ENSG00000253854 | lncRNA | 0.6106804 | 6.39E-14 | 0.58125782 | 0 |
| AC253536.3 | ENSG00000232545 | lncRNA | 0.61852364 | 2.47E-14 | 0.64394861 | 9.412E-16 |
| RBM5-AS1 | ENSG00000281691 | lncRNA | 0.58377586 | 1.37E-12 | 0.59319142 | 4.847E-13 |
| AL132989.1 | ENSG00000258682 | lncRNA | 0.62303927 | 1.41E-14 | 0.56449769 | 1.046E-11 |
| AC139887.2 | ENSG00000249592 | lncRNA | 0.61770151 | 2.74E-14 | 0.61647599 | 0 |
| AC073569.2 | ENSG00000258048 | lncRNA | 0.62308103 | 1.41E-14 | 0.61875004 | 2.405E-14 |
| AC018752.1 | ENSG00000250015 | lncRNA | 0.56254395 | 1.28E-11 | 0.5855081 | 1.136E-12 |
| Z94721.2 | ENSG00000272980 | lncRNA | 0.55537665 | 2.61E-11 | 0.6079756 | 8.816E-14 |
| AC107021.1 | ENSG00000243415 | lncRNA | 0.55150546 | 3.82E-11 | 0.56344633 | 1.164E-11 |
| AC245060.2 | ENSG00000234630 | lncRNA | 0.59494642 | 3.98E-13 | 0.64348524 | 1.002E-15 |
| AC005034.5 | ENSG00000271452 | lncRNA | 0.63114711 | 5.07E-15 | 0.57437025 | 0 |
| Z68871.1 | ENSG00000239407 | lncRNA | 0.67317574 | 1.47E-17 | 0.64752885 | 5.794E-16 |
| AL133406.2 | ENSG00000231628 | lncRNA | 0.58690367 | 9.75E-13 | 0.59029842 | 6.698E-13 |
| ZNF451-AS1 | ENSG00000226803 | lncRNA | 0.64279546 | 1.1E-15 | 0.67126053 | 0 |
| HCG18 | ENSG00000231074 | lncRNA | 0.67160026 | 1.86E-17 | 0.6330887 | 0 |
| CR936218.1 | ENSG00000262372 | lncRNA | 0.61306605 | 4.8E-14 | 0.57964294 | 2.145E-12 |
| ITPR1-DT | ENSG00000231249 | lncRNA | 0.55485797 | 2.75E-11 | 0.61404973 | 4.265E-14 |
| TRAM2-AS1 | ENSG00000225791 | lncRNA | 0.57510166 | 3.48E-12 | 0.56995266 | 0 |
| TGFB2-AS1 | ENSG00000232480 | lncRNA | 0.67360273 | 1.38E-17 | 0.68028878 | 4.972E-18 |
| AC015922.2 | ENSG00000265519 | lncRNA | 0.65082195 | 3.69E-16 | 0.62737991 | 8.195E-15 |
| C5orf66-AS1 | ENSG00000249082 | lncRNA | -0.6142912 | 4.14E-14 | -0.5862296 | 1.05E-12 |
| AP000786.1 | ENSG00000255893 | lncRNA | 0.66538058 | 4.67E-17 | 0.68437727 | 2.628E-18 |
| HCG15 | ENSG00000227214 | lncRNA | 0.63193616 | 4.58E-15 | 0.62869691 | 0 |
| NADK2-AS1 | ENSG00000245711 | lncRNA | 0.59331807 | 4.78E-13 | 0.62487624 | 1.124E-14 |
| AL662791.1 | ENSG00000244349 | lncRNA | 0.61041984 | 6.59E-14 | 0.58794547 | 0 |
| AC105339.2 | ENSG00000252690 | lncRNA | 0.57250955 | 4.57E-12 | 0.57948162 | 2.183E-12 |
| MAP3K14-AS1 | ENSG00000267278 | lncRNA | 0.64384383 | 9.55E-16 | 0.64618024 | 0 |
| NKILA | ENSG00000278709 | lncRNA | 0.56238196 | 1.3E-11 | 0.5576995 | 0 |
| AL513314.2 | ENSG00000276997 | lncRNA | 0.58206496 | 1.65E-12 | 0.5567557 | 2.279E-11 |
| NPTN-IT1 | ENSG00000281183 | lncRNA | 0.67673328 | 8.58E-18 | 0.67816418 | 6.896E-18 |
| LINC01719 | ENSG00000233396 | lncRNA | 0.56727356 | 7.87E-12 | 0.62466626 | 0 |
| AC034102.6 | ENSG00000258317 | lncRNA | 0.60049112 | 2.11E-13 | 0.67899975 | 6.066E-18 |
| AP000487.1 | ENSG00000246889 | lncRNA | 0.58045085 | 1.97E-12 | 0.65420928 | 0 |
| AC010976.1 | ENSG00000231731 | lncRNA | 0.63329716 | 3.84E-15 | 0.62077105 | 0 |
| AC109460.2 | ENSG00000260853 | lncRNA | 0.66754022 | 3.4E-17 | 0.62809161 | 7.487E-15 |
| AC007938.2 | ENSG00000270823 | lncRNA | 0.57763909 | 2.66E-12 | 0.61297487 | 4.855E-14 |
| AC018695.4 | ENSG00000270184 | lncRNA | 0.65317086 | 2.66E-16 | 0.63016317 | 5.749E-15 |
| LINC00624 | ENSG00000278811 | lncRNA | 0.64994253 | 4.16E-16 | 0.70551246 | 8.193E-20 |
| AC067852.3 | ENSG00000267632 | lncRNA | 0.5949874 | 3.96E-13 | 0.60816436 | 8.622E-14 |
| AC022211.2 | ENSG00000263843 | lncRNA | 0.56101418 | 1.49E-11 | 0.57250003 | 0 |
| AC108010.1 | ENSG00000242588 | lncRNA | 0.68483442 | 2.45E-18 | 0.65707459 | 1.539E-16 |
| GORAB-AS1 | ENSG00000231407 | lncRNA | 0.56548661 | 9.45E-12 | 0.61278714 | 0 |
| LINC00216 | ENSG00000279636 | lncRNA | 0.5931206 | 4.89E-13 | 0.62197482 | 1.615E-14 |
| AC005519.1 | ENSG00000258559 | lncRNA | 0.57128243 | 5.19E-12 | 0.55550271 | 2.58E-11 |
| AL596325.2 | ENSG00000272574 | lncRNA | 0.55131781 | 3.89E-11 | 0.55591957 | 0 |
| PABPC4-AS1 | ENSG00000228060 | lncRNA | 0.59059802 | 6.48E-13 | 0.5880018 | 8.639E-13 |
| AC017074.1 | ENSG00000227359 | lncRNA | 0.59344834 | 4.71E-13 | 0.63919009 | 1.776E-15 |
| ZNF8-ERVK3-1 | ENSG00000267216 | lncRNA | 0.57018036 | 5.83E-12 | 0.59537571 | 3.789E-13 |
| AC010761.4 | ENSG00000265474 | lncRNA | 0.63811567 | 2.05E-15 | 0.62314102 | 1.397E-14 |
| AC108058.1 | ENSG00000238273 | lncRNA | 0.56401781 | 1.1E-11 | 0.61893061 | 2.353E-14 |
| AC099518.6 | ENSG00000278389 | lncRNA | -0.6019412 | 1.79E-13 | -0.597619 | 0 |
| C1RL-AS1 | ENSG00000205885 | lncRNA | 0.57471812 | 3.62E-12 | 0.56354233 | 0 |
| LINC00630 | ENSG00000223546 | lncRNA | 0.58860911 | 8.08E-13 | 0.56752783 | 0 |
| AC022784.5 | ENSG00000254340 | lncRNA | 0.57240251 | 4.62E-12 | 0.55424779 | 2.92E-11 |
| AC132872.3 | ENSG00000275888 | lncRNA | 0.62016182 | 2.02E-14 | 0.57520863 | 0 |
| AP000442.1 | ENSG00000255139 | lncRNA | 0.64209014 | 1.21E-15 | 0.60524177 | 0 |
| AL442125.2 | ENSG00000276916 | lncRNA | 0.57586109 | 3.21E-12 | 0.57983259 | 2.102E-12 |
| AC138028.4 | ENSG00000260121 | lncRNA | 0.65556339 | 1.9E-16 | 0.61935871 | 0 |
| AL157838.1 | ENSG00000197670 | lncRNA | 0.60206011 | 1.76E-13 | 0.67340806 | 0 |
| SUCLG2-AS1 | ENSG00000241316 | lncRNA | 0.56324985 | 1.19E-11 | 0.63563064 | 2.835E-15 |
| LINC01133 | ENSG00000224259 | lncRNA | -0.5744436 | 3.73E-12 | -0.5866557 | 0 |
| AC109587.1 | ENSG00000244513 | lncRNA | 0.5679404 | 7.35E-12 | 0.56857257 | 0 |
| AC009812.4 | ENSG00000260317 | lncRNA | 0.5861121 | 1.06E-12 | 0.60925307 | 0 |
| AC093110.1 | ENSG00000238018 | lncRNA | 0.70274652 | 1.31E-19 | 0.66571836 | 4.444E-17 |
| LAMC1-AS1 | ENSG00000224468 | lncRNA | 0.66680238 | 3.79E-17 | 0.64026184 | 1.541E-15 |
| AC138393.3 | ENSG00000278467 | lncRNA | 0.61376907 | 4.41E-14 | 0.58873638 | 7.966E-13 |
| AP001094.2 | ENSG00000265413 | lncRNA | 0.57280556 | 4.43E-12 | 0.61082974 | 6.28E-14 |
| AL049840.2 | ENSG00000246451 | lncRNA | 0.62109642 | 1.8E-14 | 0.59129252 | 0 |
| RHOQ-AS1 | ENSG00000250116 | lncRNA | 0.64647906 | 6.68E-16 | 0.71065166 | 3.364E-20 |
| AC114763.1 | ENSG00000228043 | lncRNA | 0.57036714 | 5.71E-12 | 0.61455119 | 4.014E-14 |
| AL109614.1 | ENSG00000276603 | lncRNA | 0.62556408 | 1.03E-14 | 0.59432762 | 4.265E-13 |
| AP003392.1 | ENSG00000254428 | lncRNA | 0.67693865 | 8.32E-18 | 0.64727697 | 5.997E-16 |
| STARD4-AS1 | ENSG00000246859 | lncRNA | 0.59639676 | 3.37E-13 | 0.6264204 | 0 |
| AL391121.1 | ENSG00000272933 | lncRNA | 0.6164707 | 3.18E-14 | 0.59482659 | 0 |
| AC037459.2 | ENSG00000253200 | lncRNA | 0.62075836 | 1.88E-14 | 0.56378094 | 0 |
| AP000759.1 | ENSG00000255320 | lncRNA | 0.56831924 | 7.06E-12 | 0.57215823 | 0 |
| ABALON | ENSG00000281376 | lncRNA | 0.62856025 | 7.05E-15 | 0.65567089 | 1.876E-16 |
| AL354733.3 | ENSG00000235298 | lncRNA | 0.64985357 | 4.21E-16 | 0.61400856 | 4.286E-14 |
| AL365436.2 | ENSG00000232536 | lncRNA | 0.57530855 | 3.4E-12 | 0.58949464 | 7.324E-13 |
| AL049840.5 | ENSG00000269940 | lncRNA | 0.6432733 | 1.03E-15 | 0.60944009 | 0 |
| AL606534.1 | ENSG00000227230 | lncRNA | 0.62898296 | 6.68E-15 | 0.64279406 | 1.099E-15 |

**Supplementary Table 2: Selected 63 lncRNAs related to expression of ZNF281 in PAAD**

| **Supplementary Table 1: Selected 63 lncRNAs related to expression of ZNF281 in PAAD** | | | | | | |
| --- | --- | --- | --- | --- | --- | --- |
| gene_name | gene_id | gene_biotype | cor_pearson | p_pearson | cor_spearman | p_spearman |
| AC087286.2 | ENSG00000259367 | lncRNA | 0.79311595 | 1.7E-16 | 0.754146069 | 3.14E-14 |
| DNM3OS | ENSG00000230630 | lncRNA | 0.72981656 | 5.2E-13 | 0.703487592 | 0 |
| CLMAT3 | ENSG00000249035 | lncRNA | 0.60610306 | 2.1E-08 | 0.754082042 | 3.17E-14 |
| LINC01415 | ENSG00000267325 | lncRNA | 0.71663862 | 2.1E-12 | 0.750157195 | 5.09E-14 |
| AL139147.1 | ENSG00000248458 | lncRNA | 0.73392276 | 3.3E-13 | 0.73348017 | 3.47E-13 |
| AC112721.2 | ENSG00000222032 | lncRNA | 0.78694742 | 4.1E-16 | 0.806384713 | 2.14E-17 |
| AC108463.2 | ENSG00000230499 | lncRNA | 0.76732656 | 6E-15 | 0.744869215 | 0 |
| AC004160.1 | ENSG00000230333 | lncRNA | 0.67665007 | 9.3E-11 | 0.725592938 | 8.2E-13 |
| LINC00654 | ENSG00000205181 | lncRNA | 0.7871431 | 4E-16 | 0.799027498 | 0 |
| LINC00578 | ENSG00000228221 | lncRNA | 0.80183665 | 4.4E-17 | 0.800435949 | 0 |
| SPATA13 | ENSG00000228741 | lncRNA | 0.71653663 | 2.1E-12 | 0.753271237 | 3.5E-14 |
| AC006033.2 | ENSG00000272908 | lncRNA | 0.70836555 | 4.9E-12 | 0.737900217 | 2.12E-13 |
| AL590428.1 | ENSG00000231652 | lncRNA | 0.66383253 | 2.8E-10 | 0.724860612 | 8.87E-13 |
| AC092376.2 | ENSG00000277954 | lncRNA | 0.76657697 | 6.6E-15 | 0.751073105 | 0 |
| MSC-AS1 | ENSG00000235531 | lncRNA | 0.84985638 | 7.2E-21 | 0.830918846 | 0 |
| AC005288.1 | ENSG00000266469 | lncRNA | 0.71444322 | 2.6E-12 | 0.753789403 | 0 |
| AC009093.1 | ENSG00000259807 | lncRNA | 0.80819949 | 1.6E-17 | 0.763017799 | 1.04E-14 |
| AC108449.2 | ENSG00000259366 | lncRNA | 0.70269005 | 8.5E-12 | 0.702816901 | 0 |
| NORAD | ENSG00000260032 | lncRNA | 0.73477309 | 3E-13 | 0.709892689 | 0 |
| AC026356.1 | ENSG00000274964 | lncRNA | 0.75115451 | 4.5E-14 | 0.701274313 | 0 |
| AC106786.1 | ENSG00000223652 | lncRNA | 0.79752176 | 8.6E-17 | 0.767166935 | 6.11E-15 |
| AC112721.1 | ENSG00000222022 | lncRNA | 0.71558601 | 2.3E-12 | 0.799585075 | 6.23E-17 |
| AL031985.3 | ENSG00000260920 | lncRNA | 0.76536132 | 7.7E-15 | 0.732930919 | 0 |
| MYOSLID | ENSG00000229647 | lncRNA | 0.70206639 | 9E-12 | 0.751565657 | 4.3E-14 |
| BAALC-AS1 | ENSG00000247081 | lncRNA | 0.78745301 | 3.8E-16 | 0.799966465 | 0 |
| AC073046.1 | ENSG00000235499 | lncRNA | 0.73221907 | 4E-13 | 0.7119778 | 3.38E-12 |
| CASC15 | ENSG00000272168 | lncRNA | 0.78990014 | 2.7E-16 | 0.768410463 | 0 |
| AC099850.3 | ENSG00000265415 | lncRNA | 0.70903003 | 4.5E-12 | 0.705533199 | 0 |
| AL691447.2 | ENSG00000232063 | lncRNA | 0.73648008 | 2.5E-13 | 0.785897546 | 4.76E-16 |
| LNCSRLR | ENSG00000240032 | lncRNA | 0.74748544 | 7E-14 | 0.767535846 | 5.82E-15 |
| AL353593.1 | ENSG00000269934 | lncRNA | 0.79391917 | 1.5E-16 | 0.822961166 | 1.31E-18 |
| AC107021.1 | ENSG00000243415 | lncRNA | 0.71217674 | 3.3E-12 | 0.743669723 | 1.09E-13 |
| HCG18 | ENSG00000231074 | lncRNA | 0.75262584 | 3.8E-14 | 0.724010731 | 0 |
| AC015922.2 | ENSG00000265519 | lncRNA | 0.62754081 | 4.7E-09 | 0.75295104 | 0 |
| BX322234.1 | ENSG00000226445 | lncRNA | 0.77744522 | 1.6E-15 | 0.754996647 | 0 |
| AC009318.3 | ENSG00000274315 | lncRNA | 0.7214746 | 1.3E-12 | 0.706270959 | 0 |
| AC068473.4 | ENSG00000267409 | lncRNA | 0.7086333 | 4.7E-12 | 0.700488345 | 1.05E-11 |
| MBNL1-AS1 | ENSG00000229619 | lncRNA | 0.64436532 | 1.3E-09 | 0.746613011 | 0 |
| HCG11 | ENSG00000228223 | lncRNA | 0.7826784 | 7.5E-16 | 0.738061704 | 0 |
| LYRM4-AS1 | ENSG00000272142 | lncRNA | 0.74573144 | 8.6E-14 | 0.731120054 | 0 |
| AC107021.2 | ENSG00000261051 | lncRNA | 0.71343451 | 2.9E-12 | 0.705231388 | 0 |
| MYHAS | ENSG00000272975 | lncRNA | 0.70410804 | 7.4E-12 | 0.768871157 | 4.89E-15 |
| AP001189.1 | ENSG00000236304 | lncRNA | 0.73276466 | 3.8E-13 | 0.736955064 | 0 |
| AC004846.1 | ENSG00000258376 | lncRNA | 0.69790456 | 1.3E-11 | 0.724111335 | 0 |
| LINC01614 | ENSG00000230838 | lncRNA | 0.83224204 | 2.4E-19 | 0.831261161 | 2.9E-19 |
| GORAB-AS1 | ENSG00000231407 | lncRNA | 0.6640564 | 2.7E-10 | 0.712206573 | 0 |
| AC079298.3 | ENSG00000280241 | lncRNA | 0.80269041 | 3.8E-17 | 0.803488188 | 3.39E-17 |
| AC005291.2 | ENSG00000273388 | lncRNA | 0.72880125 | 5.8E-13 | 0.72037566 | 1.42E-12 |
| Z99289.1 | ENSG00000237234 | lncRNA | 0.67638864 | 9.6E-11 | 0.715493604 | 2.36E-12 |
| LINC00630 | ENSG00000223546 | lncRNA | 0.7139123 | 2.8E-12 | 0.743024816 | 0 |
| HAS2-AS1 | ENSG00000248690 | lncRNA | 0.69807086 | 1.3E-11 | 0.737089202 | 0 |
| LINC00702 | ENSG00000233117 | lncRNA | 0.75739968 | 2.1E-14 | 0.723977197 | 0 |
| LINC01094 | ENSG00000251442 | lncRNA | 0.78002866 | 1.1E-15 | 0.739369551 | 0 |
| AP001434.1 | ENSG00000226012 | lncRNA | 0.74904918 | 5.8E-14 | 0.704330669 | 7.22E-12 |
| WT1-AS | ENSG00000183242 | lncRNA | 0.63870516 | 2.1E-09 | 0.702438043 | 8.68E-12 |
| AP001189.3 | ENSG00000254810 | lncRNA | 0.78293925 | 7.2E-16 | 0.771998659 | 0 |
| AC022034.1 | ENSG00000237807 | lncRNA | 0.72971598 | 5.3E-13 | 0.741750503 | 0 |
| AP003119.3 | ENSG00000261578 | lncRNA | 0.74192928 | 1.3E-13 | 0.740073776 | 0 |
| OIP5-AS1 | ENSG00000247556 | lncRNA | 0.83817007 | 7.7E-20 | 0.818477532 | 0 |
| AC114284.1 | ENSG00000248927 | lncRNA | 0.78257309 | 7.6E-16 | 0.797054372 | 9.18E-17 |
| WNT5A-AS1 | ENSG00000244586 | lncRNA | 0.82589461 | 7.8E-19 | 0.840034878 | 5.36E-20 |
| VCAN-AS1 | ENSG00000249835 | lncRNA | 0.75554058 | 2.7E-14 | 0.759887208 | 1.55E-14 |
| USP46-DT | ENSG00000248866 | lncRNA | 0.7350238 | 2.9E-13 | 0.737826962 | 0 |

**Supplementary Table 3: Selected 41 lncRNAs related to expression of ZNF281 in STAD**

| **Supplementary Table 3: Selected 41 lncRNAs related to expression of ZNF281 in STAD** | | | | | | |
| --- | --- | --- | --- | --- | --- | --- |
| gene_name | gene_id | gene_biotype | cor_pearson | p_pearson | cor_spearman | p_spearman |
| AL355574.1 | ENSG00000238058 | lncRNA | 0.5625926 | 6.76E-14 | 0.56446775 | 0 |
| CTBP1-DT | ENSG00000196810 | lncRNA | 0.551462 | 2.61E-13 | 0.56040002 | 0 |
| AL049840.4 | ENSG00000269910 | lncRNA | 0.6106389 | 1.07E-16 | 0.60766612 | 0 |
| ERICD | ENSG00000280303 | lncRNA | 0.5872206 | 2.83E-15 | 0.60988133 | 0 |
| AC005288.1 | ENSG00000266469 | lncRNA | 0.7304249 | 2.78E-26 | 0.70629095 | 0 |
| AC005332.6 | ENSG00000278730 | lncRNA | 0.5736905 | 1.67E-14 | 0.61382817 | 0 |
| NORAD | ENSG00000260032 | lncRNA | 0.6993555 | 2.43E-23 | 0.70582515 | 0 |
| AL118506.1 | ENSG00000268858 | lncRNA | 0.5643552 | 5.43E-14 | 0.56320192 | 0 |
| FGD5-AS1 | ENSG00000225733 | lncRNA | 0.6494219 | 2.49E-19 | 0.62189608 | 0 |
| AC107068.1 | ENSG00000259959 | lncRNA | 0.5781844 | 9.36E-15 | 0.5892973 | 0 |
| AL162595.1 | ENSG00000177788 | lncRNA | 0.587355 | 2.78E-15 | 0.56565536 | 0 |
| STARD7-AS1 | ENSG00000204685 | lncRNA | 0.6310883 | 4.87E-18 | 0.65207698 | 0 |
| AC009041.3 | ENSG00000276931 | lncRNA | 0.5707019 | 2.45E-14 | 0.58641717 | 0 |
| EBLN3P | ENSG00000281649 | lncRNA | 0.6798513 | 1.11E-21 | 0.66332015 | 0 |
| AC099850.3 | ENSG00000265415 | lncRNA | 0.6250418 | 1.24E-17 | 0.62069425 | 0 |
| SP2-AS1 | ENSG00000234494 | lncRNA | 0.5557743 | 1.55E-13 | 0.55449753 | 0 |
| AC004943.2 | ENSG00000259768 | lncRNA | 0.5997732 | 5.06E-16 | 0.58816303 | 0 |
| AC005034.5 | ENSG00000271452 | lncRNA | 0.5686942 | 3.16E-14 | 0.5715934 | 0 |
| AC105036.3 | ENSG00000260269 | lncRNA | 0.5684614 | 3.25E-14 | 0.58047202 | 0 |
| Z68871.1 | ENSG00000239407 | lncRNA | 0.6665254 | 1.29E-20 | 0.65765234 | 0 |
| HCG18 | ENSG00000231074 | lncRNA | 0.6390123 | 1.38E-18 | 0.66051825 | 0 |
| LINC00205 | ENSG00000223768 | lncRNA | 0.5710934 | 2.33E-14 | 0.57847015 | 0 |
| TRAM2-AS1 | ENSG00000225791 | lncRNA | 0.5633997 | 6.12E-14 | 0.57970043 | 0 |
| PTOV1-AS1 | ENSG00000268006 | lncRNA | 0.5625566 | 6.79E-14 | 0.55533313 | 0 |
| NKILA | ENSG00000278709 | lncRNA | 0.5507223 | 2.85E-13 | 0.58681541 | 0 |
| AC092171.2 | ENSG00000230733 | lncRNA | 0.5654671 | 4.73E-14 | 0.59874839 | 0 |
| TMEM147-AS1 | ENSG00000236144 | lncRNA | 0.5606564 | 8.58E-14 | 0.58480999 | 0 |
| LINC01128 | ENSG00000228794 | lncRNA | 0.5743882 | 1.53E-14 | 0.58962087 | 0 |
| RNF216P1 | ENSG00000196204 | lncRNA | 0.6441581 | 5.97E-19 | 0.6337295 | 0 |
| AC005562.1 | ENSG00000214719 | lncRNA | 0.6815674 | 8.04E-22 | 0.64805547 | 0 |
| COX10-AS1 | ENSG00000236088 | lncRNA | 0.5784848 | 9E-15 | 0.56856038 | 0 |
| SNHG16 | ENSG00000163597 | lncRNA | 0.5706041 | 2.48E-14 | 0.5597351 | 0 |
| ZNF8-ERVK3-1 | ENSG00000267216 | lncRNA | 0.5872985 | 2.8E-15 | 0.58675141 | 0 |
| LINC01521 | ENSG00000213888 | lncRNA | 0.6017217 | 3.85E-16 | 0.61130717 | 0 |
| AC005034.3 | ENSG00000270696 | lncRNA | 0.6888093 | 1.99E-22 | 0.67645851 | 0 |
| LINC00630 | ENSG00000223546 | lncRNA | 0.7365652 | 6.51E-27 | 0.73139428 | 0 |
| MCM3AP-AS1 | ENSG00000215424 | lncRNA | 0.567923 | 3.48E-14 | 0.56435042 | 0 |
| ATXN1-AS1 | ENSG00000229931 | lncRNA | 0.5603002 | 8.96E-14 | 0.57888262 | 0 |
| STARD4-AS1 | ENSG00000246859 | lncRNA | 0.5625655 | 6.78E-14 | 0.56058847 | 0 |
| AP000759.1 | ENSG00000255320 | lncRNA | 0.5703024 | 2.58E-14 | 0.57168585 | 0 |
| AL049840.5 | ENSG00000269940 | lncRNA | 0.564737 | 5.18E-14 | 0.55203698 | 0 |
